# *Apilactobacillus kunkeei* releases RNA-associated membrane vesicles and proteinaceous nanoparticles

**DOI:** 10.1093/femsml/uqad037

**Published:** 2023-08-29

**Authors:** Christian Seeger, Karl Dyrhage, Kristina Näslund, Siv G E Andersson

**Affiliations:** Molecular Evolution, Department of Cell and Molecular Biology, Science for Life Laboratory, Biomedical Centre, Uppsala University, 752 36 Uppsala, Sweden; Molecular Evolution, Department of Cell and Molecular Biology, Science for Life Laboratory, Biomedical Centre, Uppsala University, 752 36 Uppsala, Sweden; Molecular Evolution, Department of Cell and Molecular Biology, Science for Life Laboratory, Biomedical Centre, Uppsala University, 752 36 Uppsala, Sweden; Molecular Evolution, Department of Cell and Molecular Biology, Science for Life Laboratory, Biomedical Centre, Uppsala University, 752 36 Uppsala, Sweden

**Keywords:** *Apilactobacillus kunkeei*, membrane vesicles, nanoparticles, proteomics, transcriptomics, electron microscopy, cryo-electron tomography

## Abstract

Extracellularly released particles, including membrane vesicles, have increasingly been recognized as important for bacterial community functions and host-interaction processes, but their compositions and functional roles differ between species and also between strains of the same species. In this study, we have determined the composition of membrane vesicles and protein particles identified in the cell-free pellets of two strains of *Apilactobacillus kunkeei*, a defensive symbiont of honeybees. The membrane vesicles were separated from the extracellular particles using density gradient ultracentrifugation. The peaks of the RNA and protein distributions were separated from each other and the highest concentration of RNA was observed in the fractions that contained the membrane vesicles while the highest protein concentration coincided with the fractions that contained extracellular particles. A comparative proteomics analysis by LC-MS/MS showed that 37 proteins with type-I signal peptides were consistently identified across the fractionated samples obtained from the cell-free pellets, of which 29 were orthologs detected in both strains. Functional predictions of the extracellular proteins revealed the presence of glycoside hydrolases, glycosyltransferases, giant proteins and peptidases. The extracellular transcriptomes mapped to a broad set of genes with a similar functional profile as the whole cell transcriptome. This study provides insights into the composition of membrane vesicles and extracellular proteins of a bee-associated symbiont.

## Teaser

The paper presents the extracellullar proteome of a lactic acid bacterium associated with honeybees.

## Introduction

Extracellular membrane vesicles are produced by cells from all domains of life and increasingly thought to play important roles for the coordination of cell population responses, interactions with phages and viruses, host immune system regulation, pathogenicity, and detoxification (reviewed in Gill et al. 2019). Although the production of extracellular vesicles was initially considered to only occur in Gram-negative bacteria, it is now well established that also Gram-positive bacteria, despite their thick peptidoglycan cell wall, produce such vesicles (Lee et al. 2009, Brown et al. 2014, Schrempf and Merling 2015, Vdovikova et al. 2017). Commonly referred to as outer membrane vesicles (OMVs) in Gram-negative bacteria, membrane vesicles (MVs) in Gram-positive bacteria and exosomes in eukaryotic cells, the extracellular membrane vesicles can transfer a wide range of cargo, including nucleic acids, proteins, and metabolites.

Numerous studies have suggested that RNA is a major component of membrane vesicles in bacteria. For example, rRNA and tRNA fragments were shown to be enriched in the MVs of *Prochlorococcus* (Biller et al. 2014) and *Escherichia coli* (Ghosal et al. 2015, Blenkiron et al. 2016). Small RNAs (sRNA) of 80–250 nucleotides were identified in the OMVs of *Pseudomonas aeruginosa* (Koeppen et al. 2016) and microRNA-size RNA of less than 70 nucleotides were detected in the MVs of several other bacteria (Choi et al. 2017, Sahr et al. 2022, Yu et al. 2022). A broad study of the composition of RNA molecules in the MVs of *Staphylococcus aureus* identified 28 sRNAs and a wide range of RNA fragments that mapped to 273 genes coding for virulence-associated factors, ribosomal proteins, transcriptional regulators and metabolic enzymes (Luz et al. 2021). Interestingly, the number and abundances of RNA fragments in the MVs were not static but changed with growth conditions (Luz et al. 2021).

Proteins have also been identified as a component of MVs, such as in *Lactobacillus* species that are commonly used in fermentation, food production and as probiotics (Grande et al. 2017, Li et al. 2017, Dean et al. 2019, 2020, Zheng et al. 2020, Hu et al. 2021). For example, more than 80 proteins, including surface proteins, glucoamylase, mucus binding protein, cell-wall hydrolase and lysozyme were identified in the MVs of *Lactobacillus acidophilus, Lactobacillus casei* and *Lactobacillus reuteri* (Dean et al. 2019). The relative fraction of proteins with signal peptides was more than 5-fold enriched in the MVs compared to the protein content of the bacterial cells, suggesting that the MVs predominantly contain secreted proteins (Dean et al. 2019). It was hypothesized that an increased expression of secreted proteins preceded the formation of vesicles, while the actual packaging of proteins into the MVs was a passive process.

Metabolites and antimicrobial compounds have also been identified in the membrane vesicles of some bacterial species. For example, the induction of a 10-kb operon for the synthesis of a putative bacteriocin in *L. acidophilus* resulted in a more than 500-fold enrichment of the bacteriocin in the MVs (Dean et al. 2020). Interestingly, molecules delivered via the MVs of *L. plantarum* have been shown to enhance host immune responses against vancomycin-resistant enterococci, suggesting that they may serve a role in host pathogen defense (Li et al. 2017).

The mechanisms whereby the vesicles are formed have not been entirely elucidated. However, several stressors that trigger the formation of these nanoparticles of about 20–500 nm have been proposed, such as imbalanced cell wall biosynthesis, explosive cell lysis and bubbling cell death (Toyofuku et al. 2019). Prophage-induced cell wall damage have been shown to promote extracellular vesicle production in *Bacillus subtilis* and *Lactococcus lactis* (Toyofuku et al. 2017, Liu et al. 2021), as has also antibiotic-induced cell stress in *Stenotrophomonas maltophilia* (Devos et al. 2017). Besides, MVs have also been shown to be produced under basal growth conditions without the use of external stressors (Rodriguez and Kuehn 2020, Luz et al. 2021).

*Apilactobacillus kunkeei* (formerly called *Lactobacillus kunkeei*) colonizes the honey crop and food products of honeybees and is thought to protect the bees from infections with bee pathogens (Vásquez et al. 2012, Butler et al. 2013, Olofsson et al. 2014). Different strains of these bacteria grow rapidly with generation times of 35 to 60 minutes under laboratory conditions and have genome sizes of about 1.5 Mb (Dyrhage et al. 2022). Previous studies of the secretome of *A. kunkeei* strain Fhon2 identified 24 secreted proteins (Butler et al. 2013), most of which are encoded by genes located near the origin of replication (Tamarit et al. 2015). It was hypothesized that some of these may confer antimicrobial functions (Butler et al. 2013), although this has not been experimentally demonstrated.

It has however been shown that *A. kunkeei* produces kunkecin A, a nisin-like bacteriocin, which has a strong inhibitory activity against the bee pathogen *Melissococcus plutonius* (Zendo et al. 2020). The gene cluster for kunkecin A biosynthesis is located on a 19.5 kb plasmid and can thereby be shared within the population. A comparative genomics study of more than 100 isolates from several beehives showed that the plasmid was broadly present in isolates obtained from one of the hives (Dyrhage et al. 2022). Based on these results, it was hypothesized that *A. kunkeei* serve a role as a defensive symbiont of honeybees.

Because of its antimicrobial activities and viability in solutions of high sugar concentrations, it is considered that *A. kunkeei* may be used as a complement in fruit preparations given to hospitalized and immunocompromised patients (Vergalito et al. 2020). The MVs of *A. kunkeei* can potentially be used for the delivery of engineered antimicrobials, vaccines or probiotics. It is therefore of general interest to characterize the particles secreted by *A. kunkeei* under normal growth conditions and determine their composition.

Here, we have performed a comprehensive study of secreted particles and membrane vesicles in *A. kunkeei*. For the purpose of this study, we have used the rapidly growing and plasmid-less *A. kunkeei* strains A1401 and A0901, which were isolated from the honey-crop of honeybees sampled from the island Åland in the Baltic Sea (Dyrhage et al. 2022). The genomes are very similar overall, but in comparison to A1401, strain A0901 contained a predicted prophage (Dyrhage et al. 2022). We have used electron microscopy techniques to examine the structures of the secreted particles, density-gradient ultracentrifugation to separate the MVs from other types of secreted particles, along with transcriptomics and proteomics to identify their molecular contents. The results are discussed in the context of previously proposed models for the role that secreted proteins and membrane vesicles play for bacterial communities and their hosts.

## Materials and methods

### Study design and workflow

**Figure 1.**
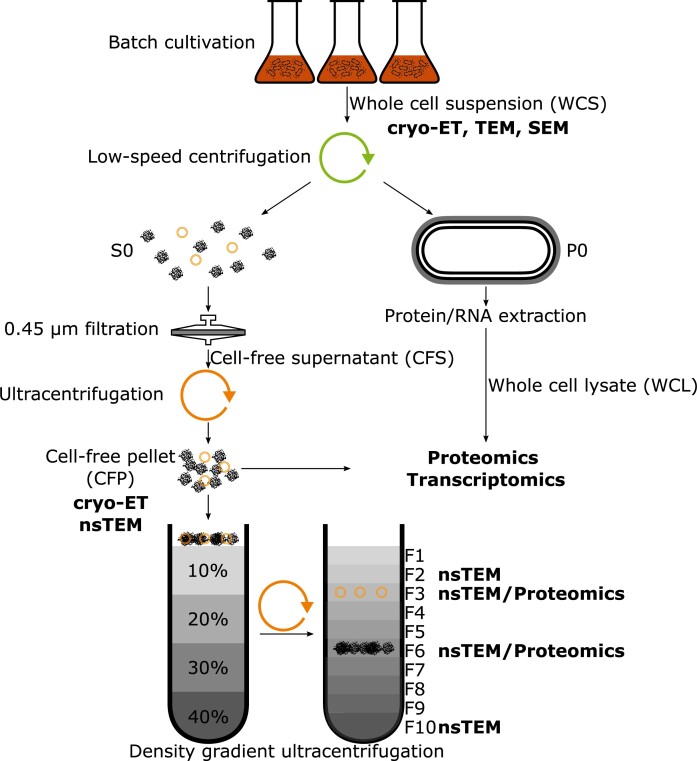
Workflow for isolation and characterization of secreted particles, CFP and WCL from *A. kunkeei*. After batch-cultivation, isolation of CFPs containing ECPs and MVs is based on low-speed centrifugation, filtration and ultracentrifugation. For further analysis, the crude CFP was fractionated by DGUC using an Optiprep gradient. For comparative analysis, pellet P0 was processed to obtain the WCL. The techniques for analysis of the different fractions are indicated.

A summary of the methods used in this study is shown in Fig. 1. We cultivated *A. kunkeei* strains A0901 and A1401, which grow with generation times of ≈35 min under laboratory conditions (Dyrhage et al. 2022). Their cell surface structures were first examined by cryo-electron tomography (cryo-ET), Transmission Electron Tomography (TEM) and Scanning Electron Tomography (SEM) at exponential growth phase. Next, we pelleted the bacterial cells by low-speed centrifugation and isolated secreted material smaller than 0.45 μm from the supernatant by filtration and ultracentrifugation. The secreted material in the cell-free pellet (CFP) was examined by cryo-ET and negative strain TEM (nsTEM). We separated the particles in the CFP using density gradient ultracentrifugation. Finally, we analyzed the composition of the secreted material in the CFPs by collecting proteomics and transcriptomics data. For comparison, we also collected proteomics and transcriptomics data from whole cell lysates (WCL) obtained after the first low-speed centrifugation step.

### Isolation of whole cell lysate and cell-free supernatant

The *A. kunkeei* strains A0901 and A1401 were cultivated in MRS-medium (Sigma Aldrich) according to (de Man et al. 1960) supplemented with 0.5% (w/v) D-Fructose (Sigma Aldrich) and 0.1% (v/v) Tween-80 (Sigma Aldrich), in the following referred to as fMRS. Batch-cultivation was performed using biological triplicates at 35°C and 5% CO_2_ in batch-cultures of 100–150 mL fMRS medium and the cells were harvested during exponential growth phase at OD600 ≈ 0.3.

After harvesting, cells and large debris were separated from the supernatant by low-speed centrifugation at 4 500 x g for 10 min at 4°C (‘P0’). To obtain samples from the WCL fraction, pellets P0 were washed twice in HyClone Hypure Water, Molecular Biology Grade (Cytiva) before being re-suspended in 25 mM Tris, pH 8.0, 6 M urea, 1x Sigma Fast protease inhibitors (Sigma Aldrich) to achieve a cell concentration corresponding to OD600 ≈ 20. Cells were lysed by sonication using a Vibra-Cell VCX 130 sonicator by repeated 10 s pulses (10–15 cycles) followed by 10 s breaks using a 2 mm probe and 30–40% amplitude (on ice). After sonication, the cleared cell suspensions were centrifuged at 17 000 x g for 10 min at room temperature and the supernatant was carefully recovered for SDS-PAGE and proteomics analysis.

To obtain samples from the cell-free supernatant (CFS), any remaining cells and debris were first removed from the crude supernatant (‘S0’) by filtering through 0.45 μm cellulose membrane filters connected to a 50 mL syringe. The cell-free filtrate (‘F1’) was subjected to ultracentrifugation at 150 000 x g for 2 h at 4°C using a 45Ti rotor in an Optima XPN-100 ultracentrifuge (Beckman). The supernatant (‘S2’) was carefully decanted and the small and translucent CFPs were allowed to air-dry for ≈ 5 min before being re-suspended in 25 mM Tris, 6 M urea, pH 8.0 for proteomic analysis. For complementary analysis using nsTEM and Nanoparticle Tracking Analysis (NTA), CFPs were re-suspended in 10 mM Tris, pH 7.5 and for cryo-ET in 1x PBS. Those methods are described in more detail below. To assess the complete removal of whole cells, 100-500 μl aliquots of filtrate F1 were plated on fMRS agar plates and incubated over night at 35°C, 5% CO_2_ along with the remaining 10-20 ml of filtrate F1.

### Separation of crude cell-free pellets by density gradient ultracentrifugation

Optiprep gradients were used for density-gradient ultracentrifugation (DGUC) of CFP preparations from *A. kunkeei* A1401 and A0901. For this, discontinuous gradients were prepared by overlayering 500 μl of Optiprep (Sigma Aldrich) solution (40%, 30%, 20%, 10% in 25 mM Tris, pH 7.4, 0.25 M Sucrose). The gradients were stored at 4°C for 60 min and 100 μL of three independent S0 CFP preparations, re-suspended in 25 mM Tris, pH 7.4, 0.25 M sucrose, were carefully placed on top of each gradient. Ultracentrifugation was performed in a Sorvall RC M150 GX centrifuge (Thermo Fisher Scientific) using a S55-S swinging-bucket rotor (Thermo Fisher Scientific) for 4 h at 216 000 x g (4°C). Fractions of 200 μL were carefully collected from top-to-bottom (F1/top–F10/bottom) and stored at 4°C. Fractions were analyzed by SDS-PAGE and nsTEM. Particle concentrations were estimated by NTA, protein concentration by Bradford (Thermo Fisher Scientific), absolute RNA concentration by the Qubit RNA HS assay (Life Technologies), relative RNA concentration by SYTO RNASelect (Life Technologies) and relative membrane abundance using the lipophilic dye FM 4–64.

### Nanoparticle tracking analysis

The particle concentration and size distribution (based on hydrodynamic radius) of the CFP preparations were analyzed by NTA on a NanoSight LM14 instrument (Malvern Panalytical, λ = 405 nm and λ = 532 nm). Particles of each sample were recorded for 5 × 30 s and quantified using the NanoSight NTA software v3.4.

### Nuclease protection assay

To investigate the localization of the RNA within the secreted particles, we performed nuclease protection assays on crude CFP preparations of *A. kunkeei* A10901 and A1401. The assays were performed using biological triplicate samples from each strain. After diluting the CFP preparations 10-fold in 10 mM Tris, samples were split into a control group (‘C’) and a treated group (‘PS’). The treated group was first subjected to proteinase K treatment (56°C, 60 min) at a final enzyme concentration of approximately 60 mAU/mL. In a second step, SDS was added to a final concentration of 1% (v/v), followed by incubation at 56°C for 30 min. The concentration of the control samples was adjusted accordingly using 10 mM Tris and incubated at the same temperatures as the treated sample. Total RNA concentration was measured using the Qubit RNA HS assay before and after addition of RNAse (final concentration 50 μg/mL, 37°C, 90 min).

### Electron microscopy

TEM, nsTEM and SEM were performed at the electron microscopy unit, EMiL, at Karolinska Institutet, Huddinge, Sweden, essentially as described in (Seeger et al. 2017, Mahajan et al. 2020). For TEM (*A. kunkeei* strain A1401) and SEM analysis (*A. kunkeei* strain A1401 and A0901, bacteria were grown in fMRS medium until log-phase (OD600 0.2–0.6) and 1 mL cell suspension was gently pelleted by centrifugation at 500 x g for 2 min at room temperature. After removing the supernatant, the cells were fixed in 1 mL fixation solution (10 mM Phosphate buffer, pH 7.4, 2% glutaraldehyde, 1% formaldehyde) for 15 min at room temperature and stored at 4°C until further processing. For nsTEM analysis of CFPs from *A. kunkeei* strains A1401 and A0901, particles were isolated as described above and processed as described in (Mahajan et al. 2020). Size distributions were generated by particle analysis in Fiji/ImageJ (Schindelin et al. 2012, Schneider et al. 2012).

### Cryo-transmission electron tomography

Cryo-ET was performed on crude CFP preparations and whole cells obtained from *A. kunkeei* strains A1401. For crude CFP preparations from *A. kunkeei* strain A1401, R2/2 200 mesh grids with a 2 nm carbon support (QuantiFoil) were glow-discharged (20 mA 60 sec) on a Quorum GloQube. 10 nm protein-A gold fiducials (Aurion) were resuspended and mixed properly with the isolated particles at a 1:10 ratio. 3 μL of this mixture were applied onto grids before plunge-freezing into liquid ethane in a Vitrobot Mark IV robot (FEI/Thermo Fisher Scientific) operated at 4°C, 100% humidity and with a blot time of 5 seconds. Datasets were collected using a Talos Arctica microscope (FEI/Thermo Fisher Scientific) outfitted with a Falcon3 detector (FEI/Thermo Fisher Scientific) operated at 200 kV in nanoprobe and TEM mode, a C2 aperture size of 50 μm, an objective aperture size of 70 μm. SerialEM software (Mastronarde 2005) was used to acquire each tilt-series using a dose-symmetric tilt scheme (Hagen et al. 2017) with a range of ±60°, 2° angular increment and a target defocus of -4 to -6 μm. Each tilt-series of 61 12-frame movies was recorded in counting mode with a pixel size of 1.23 Å at a dose rate of 0.8 e^−^/px/s for 3 sec and a total dose per tilt-series of ∼95e^−^/Å^2^. The initial raw movies were aligned and dose-weight filtered using ‘alignframes’ from the IMOD package (Mastronarde and Held 2017). Tilt-series were aligned using the gold fiducial markers, and tomograms were reconstructed by weighted back-projection using programs within IMOD v4.11.6 (Mastronarde and Held 2017).

For cryo-ET of whole cells, R2/2 200 mesh grids (QuantiFoil) were glow-discharged (20 mA 60 sec) on a Quorum GloQube.10 nm protein-A gold fiducials (Aurion) were resuspended and mixed properly with the cells at a 1:2 ratio. Three microliters of this mixture were applied onto grids before plunge-freezing into liquid ethane in a Vitrobot Mark IV robot (FEI/Thermo Fisher Scientific) operated at 4°C, 100% humidity and with manual back-side blotting of 5 seconds done within the Vitrobot humidified chamber. Datasets were collected using a Titan Krios G3i microscope (FEI/Thermo Fisher Scientific) outfitted with a K3 detector and BioQuantum imaging filter (Gatan) operated at 300 kV in nanoprobe and EF-TEM mode, a C2 aperture size of 50 μm, an objective aperture size of 100 μm, and an energy filter slit width of 20 eV in Zero-Loss mode. SerialEM software was used to acquire each tilt-series using a dose-symmetric tilt scheme with a range of ±60°, 2° angular increment and a target defocus of -3 to -6 μm. Each tilt-series of 61 10-frame movies was recorded in counting mode with a pixel size of 2.11 Å at a dose rate of 15 e^−^/px/s for 0.4 s and a total dose per tilt-series of ∼83e^−^/Å^2^. The initial raw movies were aligned and dose-weight filtered using ‘alignframes’ from the IMOD package. Tilt-series were aligned using the gold fiducial markers, and tomograms were reconstructed by weighted back-projection using programs within IMOD v4.11.6 (Mastronarde and Held 2017). Size distributions were generated by particle analysis in Fiji/ImageJ (Schindelin et al. 2012, Schneider et al. 2012).

### Proteomics analysis

Reagents for SDS-PAGE were purchased from Life Technologies. For SDS-PAGE of the extracted proteins from the WCL and CFP fractions, 1x NuPAGE LDS Sample Buffer and 1x NuPAGE Sample Reducing Agent were incubated at 70°C for 10 min. About 10 μL sample and 5 μL Novex Sharp Unstained Protein Standard were loaded on a NuPAGE 4–12% Bis-Tris Protein Gel (1.0 mm) and electrophoresis was performed in 1x MOPS SDS Running buffer supplemented with NuPAGE Antioxidant for 55 min at 200 V. The gel was washed, stained and destained using SimplyBlue SafeStain based on the manufacturers microwave protocol and imaged on a ChemiDoc MP imaging system (Bio-Rad).

Biological triplicates of fractions DGUC F3 and F6 from *A. kunkeei* strains A1401 and A0901 were subjected for proteomics analysis after in-solution digestion, essentially as described previously (Seeger et al. 2021). Notably, for the F6 samples that contained a high concentration of proteins, 10 μg of proteins was used for the digestion (ca 10 μl of the sample). For the F3 samples that contained non-measurable concentration of proteins, the entire sample was used for the digestion (ca 100 μl of the sample). In addition, the gel bands from biological triplicates of both *A. kunkeei* A1401 and A0901 in the area between 110–150 kDa of fraction F6 were excised and subjected for proteomics analysis after in-gel digestion, similar as described previously (Mahajan et al. 2020).

Extracted proteins of the WCL and CFP fractions were analyzed by Label-free quantification (LFQ) by LC-MS/MS as described previously (Seeger et al. 2021). For the LFQ analyses, MS raw files were processed using MaxQuant software (Cox and Mann 2008) and the Andromeda search engine (Cox et al. 2011) against the genome sequences of *A. kunkeei* A0901 and A1401. For the qualitative analysis of the DGUC fractions, database searches were performed using the Sequest algorithm, embedded in Proteome Discoverer 1.4 (Thermo Fisher Scientific) against the genome sequences of *A. kunkeei* A0901 and *A. kunkeei* A1401 that were also used for the analysis of the crude WCL and CFP fractions. Further analysis and visualization were performed in R (R Core Team 2021) using the package ggplot (Wickham 2016,R Core Team 2021). Transmembrane proteins were predicted using Phobius v1.1 (Käll et al. 2004, 2007). Signal peptides (type I and type II) were predicted using the SignalP-5.0 Server (Almagro Armenteros et al. 2019). Functional classification was based on the Clusters of Orthologous Genes, COGs (Tatusov et al. 1997, Galperin et al. 2021).

### Transcriptomics analysis

For the transcriptomics analysis, the RNA was extracted from WCL and CFP fractions following cultivation of *A. kunkeei* strain A1401 in biological triplicates as described above. After separation of the cell pellet P0 from the crude supernatant S0, P0 was resuspended in RNAlater, stored at 4°C overnight before storage at -20°C. The cell-free pellet (CFP) was re-suspended in HyClone Hypure Water, Molecular Biology Grade (Cytiva) and stored at -20°C.

The RNA extraction was performed using the RiboPure-Bacteria Kit including DNAse I treatment (LifeTechnologies). Sequencing libraries were prepared from 500 ng total RNA using the Illumina Stranded Total RNA Prep, Ligation with Ribo-Zero Plus kit (Illumina, cat#20 040 525). The library preparation was performed according to the manufacturers’ protocol (# 1000000124514–00). Sequencing was performed on a MiSeq system using v3 sequencing chemistry (Illumina) and a paired-end 75 bp read length.

For the transcriptomics analyses, the obtained sequence reads were mapped to the genome sequence of *A. kunkeei* strain A1401 using hisat2 v2.2.1 (Kim et al. 2019). Read counts for the 1423 predicted coding sequences, including 9 predicted extrachromosomal coding sequence (Dyrhage et al. 2022), were determined using FeatureCounts v2.0.1 (Liao et al. 2014). For filtering genes with low expression levels and for allowing comparisons between genes, raw read counts were transformed to transcripts per million (TPM) (Wagner et al. 2012). Only genes with transcripts with TPM values ≥ 10 in either the CFP or the WCL fraction were retained for further analysis, similar as described previously (Srikumar et al. 2015). Differential enrichment analysis using raw read counts was performed in R using the DESeq2 method (Love et al. 2014). The standard DESeq2 workflow includes normalization of raw read counts to account for sequencing depth and compositional bias as well as correction for gene dispersion as a means to measure variation. Genes with significant differences in their abundance between the CFP and WCL fractions (adjusted p-value < 0.05) and an absolute log2-ratio larger than the standard deviation of the determined log2-ratios of the dataset were classified as differentially enriched.

## Results

The workflow of this study is schematically depicted in Fig. 1. *A. kunkeei* strains A1401 and A0901 were cultivated in fMRS medium and the bacterial cells were harvested during the exponential growth phase. We examined the morphologies of particles detected on the surfaces of the bacterial cells as well as the morphologies and composition of secreted particles and vesicles obtained from the pelleted supernatant, after removal of the bacterial cells. We also examined the proteomes and transcriptomes of the whole cell lysates (WCL) and the pellet of the cell free supernatant (CFP).

### Morphological characterization of cell surface particles

**Figure 2.**
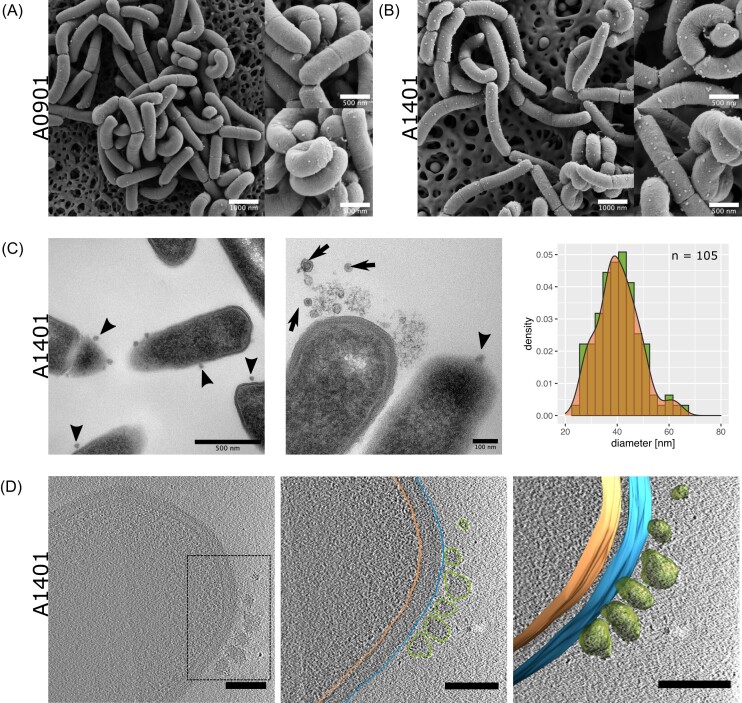
Cell surface particles of *A. kunkeei*. Particles on the surfaces of bacterial cells are shown by **(A, B)** SEM, **(C)** TEM and **(D)** cryo-ET micrographs of *A. kunkeei* strains **(A, C, D)** A1401 and **(B)** A0901. **(C)** The size distribution is based on particle measurements performed in Fiji/ImageJ based on analyses of the TEM images. **(D)** The raw (left), a segmented tomograph (middle) and the final segmentation of the cryo-ET tomogram are are shown (scale bar: 100 nm).

Images produced by SEM showed that *A. kunkeei* strains A1401 and A0901 formed regularly rod-shaped cells as well as corkscrewed cells, both of which contained particles on the cell surfaces (Fig. 2AB). The particles of *A. kunkeei* strain A1401 were examined in more detail by TEM, which showed that the majority of particles were contrast-rich with speckled surfaces (Fig. 2C). Likewise, tomography analysis based on cryo-ET of *A. kunkeei* strain A1401 showed the presence of particles on the bacterial cell surfaces, some of which were located in close vicinity to each other (Fig. 2D). The mean (median) diameter of the particles was estimated to 39.9 nm (40.5 nm) based on analyses of the TEM images (n = 105) (Fig. 2C, and to 36.2 nm (36.2 nm) based on analyses of the cryo-ET micrographs (n = 12) (Table S1).

### Morphological characterization of extracellular particles and vesicles

**Figure 3.**
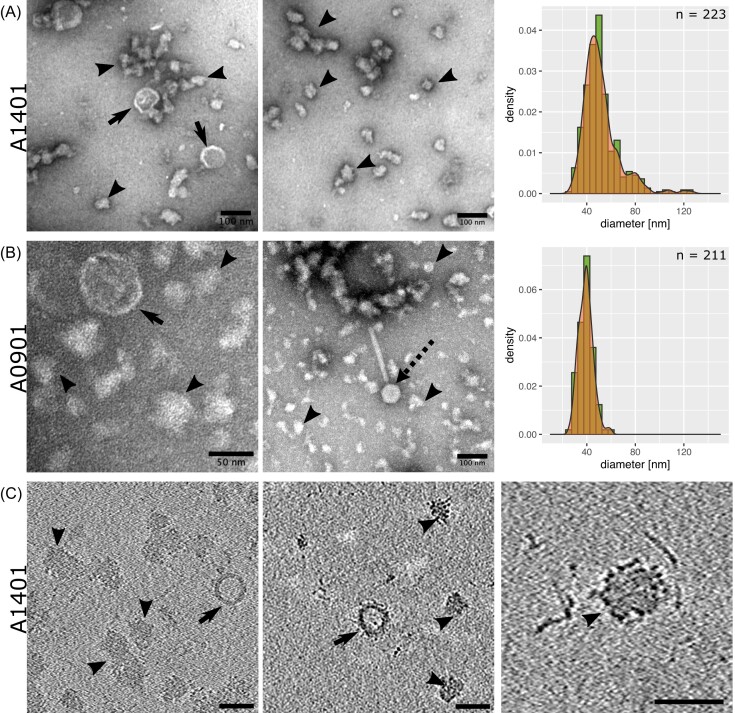
Particles secreted by *A. kunkeei*. Particles secreted by *A. kunkeei* strains **(A)** A0901 and (BC) A1401 are shown by (AB) nsTEM and **(C)** cryo-ET micrographs (scale bar: 100 nm). (AB) The size distributions were estimated from measurements of the sizes of all particles in the nsTEM images. Black arrows and arrowheads point to MVs and particles, respectively. A phage particle is also indicated.

Next, we studied the morphology of the extracellular material in the cell-free pellet, which was obtained after centrifugation of the medium in which the bacterial cells had been cultivated, and as a negative control we also pelleted the fMRS medium. The negative stain TEM micrographs of the dissolved pellets from the two *A. kunkeei* strains (Fig. 3AB) showed the presence of two morphologically distinct particle types, one of which resembled membrane vesicles (MVs) while the other type consisted of irregularly shaped particles, which we refer to as extracellular particles (ECPs). No such particles were visible in the media control samples. The micrographs also revealed the presence of a phage in *A. kunkeei* strain A0901 (Fig. 3B), consistent with a prophage in the genome of A0901 (Dyrhage et al. 2022). Cryo-ET tomography of the extracellular material obtained from strain A1401 showed MVs containing lipid bilayers separated by ≈ 4 nm as well as contrast-rich ECPs with speckled surfaces (Fig. 3C). The negative stain TEM (Fig. 3A) and cryo-ET images (Fig. 3C) indicated that some of the ECPs aggregated. The mean (median) diameter of particles that resembled ECPs in morphology in the nsTEM images were estimated to 39.1 nm (39.2) nm in strain A0901 (n = 211) (Fig. 3A, Table S1) and to 48.9 nm (52.2 nm) in strain A1401 (n = 223) (Fig. 3B, Table S1). Likewise, the cryo-ET images also showed that the ECPs were homogeneous in size (29.4–39.3 nm), with a mean (median) diameter of 39.3 (37.3 nm) in strain A1401 (n = 12) (Table S1). A few particles of variable sizes that resembled MVs were identified in the nsTEM micrographs, but they were too few to allow accurate size estimates. The cryo-ET images indicated that the MVs were of variable sizes (23–162 nm) with an estimated mean (median) diameter of 64.7 nm (46.7 nm) (n = 13),

### Biochemical and biophysical characterization of secreted particles

The particle concentrations in the CFPs derived from *A. kunkeei* strains A0901 and A1401 were determined by NTA, and the RNA and protein contents were quantified using Qubit fluorometric assays (Figure S1, Table S2). The particle, protein and RNA concentrations were consistently higher in the CFP from *A. kunkeei* strain A0901 than from strain A1401 despite similar cell density during batch-cultivation. The size distribution of the isolated particles, based on the hydrodynamic radius measured by NTA, indicated a polydisperse sample. The mode diameter (major peak in the distribution) of the secreted particles was estimated to 84 nm (n = 3) for *A. kunkeei* strain A0901 and 68 nm (n = 3) for *A. kunkeei* strain A1401 (Figure S2, Table S2). Total RNA concentrations in the crude CFP samples were measured in untreated control samples and in samples after treatment with proteinase K and SDS. No significant differences in the RNA concentrations were found between the untreated and the treated samples (Figure S3). However, the RNA concentration decreased below the detection limit in both samples after treatment with RNAse. These results suggest that the RNA is located extravesicularly and/or is only loosely associated with the particles (Figure S3).

### Separation of membrane vesicles from protein particles

In order to investigate whether the membrane vesicles and extracellular particles in the CFP could be separated, we performed density gradient ultracentrifugation of the CFP derived from *A. kunkeei* strain A1401 using an Optiprep gradient. We analyzed 10 fractions from the gradient for particle concentration, protein content, RNA concentration and relative membrane abundance. The absolute concentrations and fluorescence intensities of the different assays are summarized in Figure S4.

**Figure 4.**
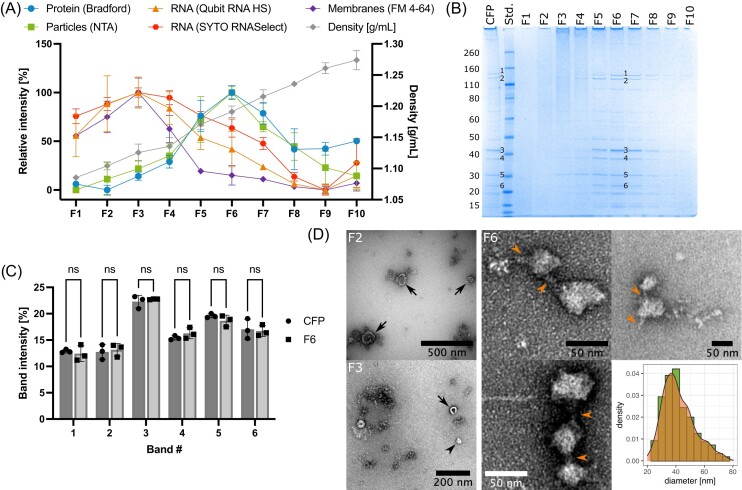
Separation of CFP components by DGUC. Crude CFP (n = 3, biological replicates) from *A. kunkeei* strain A1401 were separated by Optiprep-based DGUC into 10 fractions. **(A)** Analysis of DGUC fractions for particles (NTA), protein (Bradford), RNA (Qubit RNA HS assay, SYTO RNASelect), membranes (FM4-64) and density. Relative concentrations and intensities are shown based on average and standard deviation from three biological replicates. Corresponding absolute values are shown in Figure S3. **(B)** SDS-PAGE analysis of DGUC fractions (F1–F10) of representative replicate sample. The crude CFP sample (10x diluted) was loaded for comparison with the DGUC fractions. Novex Sharp Unstained marker was used as the molecular weight standard and the corresponding molecular weights (in kDa) are shown next to the gel. **(C)** Comparison of relative band intensities of 6 selected bands from crude CFP fraction and DGUC fraction F6. The band numbers on the x-axis correspond to the band numbers in **(B). (D)** nsTEM analysis of DGUC fractions F2, F3 and F6. Black arrows point towards MVs, orange arrowheads point towards protrusions from and connections between ECPs. The size distribution is based on particle measurements performed in Fiji/ImageJ.

The relative particle abundance (determined by NTA) across the 10 fractions correlated strongly with the protein profile (determined by Bradford). Both types of analyses showed that fractions F5 to F7 contained the majority of particles and proteins in both strain A1401 (Fig. 4A) and strain A0901 (Figure S5A). Consistently, analysis by SDS-PAGE showed the highest protein abundance in fraction F6 (Fig. 4B; Figure S5B). The sizes and intensities of the strongest bands in fraction F6 (density: 1.19 g/mL) were identical to those in the crude CFP- preparation (Fig. 4C). Bands of similar sizes were visible also in the flanking fractions F4-F5 and F7-F8, but at gradually lower intensities, in accordance with the lower total abundance of proteins in these fractions. Interestingly, the membrane fluorescence intensity across the 10 fractions correlated with the intensity profile of RNA rather than with the protein profile (FM 4–64, SYTO RNASelect and Qubit RNA HS), with the highest intensity detected in fraction F3 (density: 1.13 g/mL, Fig. 4A; Figure S5A). RNA was also detected in fractions F5 to F7, although the membrane fluorescence was lower in these fractions.

The morphologies of the particles detected in fractions F2, F3 and F6 were analyzed by nsTEM (Fig. 4D; Figure S5C). Fraction F2 and F3 contained mostly MVs and particles larger than 20 nm were only rarely observed. Vice versa, fraction F6 contained a large number of ECPs and only very few MVs. Some of the observed particles in fraction F6 appeared to be connected by a filamentous structure of approximately 6 nm in diameter. We also observed single particles that displayed small protrusions of morphologically similar structures. The size distribution of the indicated a mean (median) diameter of 42.2 nm (39.0 nm) in fraction F6 from *A. kunkeei* strain A1401 (n = 206) and of 30.3 nm (29.8 nm) in fraction F6 from strain A0901 (n= 100) (Table S1). Taken together, this suggests that the membrane vesicles can be separated from the speckled particles, and that the particles mostly consist of proteins while the RNA fractionated with the membrane vesicles.

### The extracellular proteome

To examine the composition of proteins in the CFP sample, we performed two sets of comparative proteomics studies in *A. kunkeei* strain A1401 and strain A0901.

**Figure 5.**
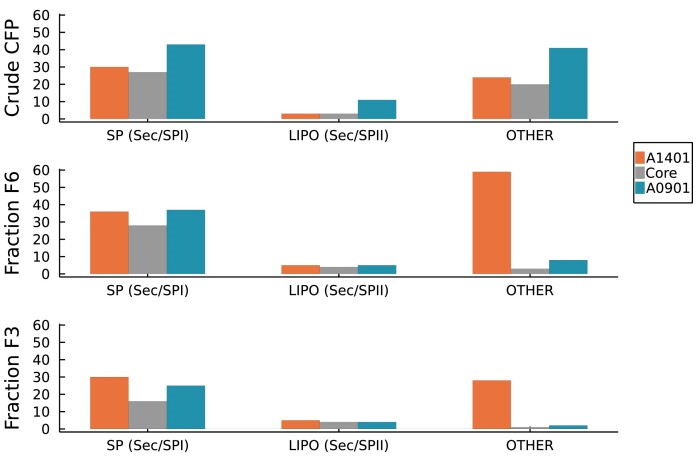
Number of protein families in the CFP proteomes. Data is shown for the CFP proteomes of *A. kunkeei* strains A1401 and A0901, and for homologs present in both strains (Core). Crude CFP refers to the number of protein families that contain proteins identified in three biological replicates in the crude CFP samples. Fraction F6 refer to the number of protein families that contain proteins identified in three biological replicates in fractions F6. Fraction F3 refers to the number of protein families that contain proteins identified in three biological replicates in fraction F3. The table shows the number of proteins in which signal peptides (SPI and SPII) were predicted by SignalP5.0. No signal peptides were predicted for proteins classified as OTHER.

In the first study, we compared the protein composition in the crude WCL and CFP samples. Analyses by SDS-PAGE showed that the band profiles differed extensively between the WCL and CFP samples for *A. kunkeei* strain A1401 (Figure S6A) and strain A0901 (Figure S6B). A pelleted media control used as a negative control showed no bands, which confirmed that the protein bands were derived from the bacterial cells and not from the media (Figure S6C). In total, 591 proteins were identified by LC-MS/MS in three biological replicates of at least one sample in *A. kunkeei* strain A1401, of which 504 proteins were only identified in the WCL sample, 9 only in the CFP sample and 45 proteins were identified in three replicates in both samples (Table S4). In *A. kunkeei* strain A0901, a total of 710 proteins were identified in three biological replicates in at least one sample, of which 566 proteins were only identified in the WCL sample, 23 were exclusively found in the CFP sample, while 61 proteins were identified in three replicates in both samples (Table S4). Type-I signal peptides were predicted for 50% of the proteins in the CFP samples (Fig. 5) as compared to only 6–7% of the proteins in the WCL samples (Table S4). In total, 44 protein families comprising 46 proteins with type-I signal peptides were detected in the CFP samples of the two strains, of which 27 families comprising 29 homologous proteins were identified in both strains (Fig. 5).

In the second study, we compared the protein compositions in the DGUC fractions F3 and F6. The concentration of proteins was estimated to 0.04–0.11 μg/μl in fraction F6, but was so low that it was unmeasurable in fraction F3 (Table S3). Consistently, the F6 and F7 fractions contained the highest abundance of proteins according to the SDS/PAGE analyses, while no or only faint bands were visible in the F1, F2 and F3 fractions (Figure S7). However, despite the different intensities of the bands, the visible band profiles were similar across lanes (Figure S7). Analyses by LC-MS/MS showed that 50 and 100 protein families contained proteins identified in 3 biological replicates in the F6 fractions of *A. kunkeei* strains A0901 and A1401, respectively. Of these, 37 proteins identified in one or both strains contained predicted type-I signal peptides (Fig. 5; Table S5). However, no peptides that matched the signal peptides of these proteins were detected in the LC/MS analyses. This suggests that the proteins with type-I signal peptides are mature and secreted although it should be cautioned that not all peptides of a protein are identified in LC/MS analyses.

The proteins identified in the F3 fractions were also examined, although the identification of protein in this fraction may be less reliable due to the overall lower abundance of proteins. Nevertheless, we found that 80% of the CFP proteins with type-I signal peptides identified in the F6 fractions in three biological replicates from both strains were also identified in the F3 fractions (Table S5). No protein was uniquely identified in three biological replicates in the F3 fraction in both strains. This suggests that the F3 and F6 fractions contain a similar set of surface-associated and secreted proteins, albeit in different abundances.

Finally, we compared the identity of proteins with type-I signal peptides across the different datasets. The comparison showed that all 37 proteins were detected in three biological replicates in the CFP sample as well as in the F3 and F6 fractions of at least one *A. kunkeei* strain, of which 29 orthologs were detected in both strains (Table S6). Functional predictions of this core set of extracellular proteins with type-I signal peptides indicated the presence of soluble proteins with glycosyl hydrolase, peptidase and nuclease enzymatic functions (Table S6). The giant proteins, which are encoded by a stretch of five co-located genes, were also part of the core set of extracellular proteins. Glycosyl hydrolases of the GH20 protein family have predicted β-hexoseamidase activity, while enzymes with the GH25 domains have predicted lysozyme activity. Notably, the core extracellular proteome also contained glycosyltransferases of the GH70 protein family, putatively involved in the synthesis of α-D-glucans, such as dextran. The sizes of two strongest bands in the SDS-PAGE gels were in the range of 110-160 kDa (Figure S7), which corresponded well with the estimated molecular weights of the 114 kDa glycosyltransferases (A0901_05 380 and A1401_04 720) and the 150-158 kDa glycosyltransferases (A0901_13 270 and A1401_12 750). In gel digestion of proteins embedded in this section of the SDS-PAGE gel confirmed that the high-intensity bands in this segment of the gel contained the GH70 family of glycosyltransferases (Table S5).

In addition, lipoprotein signal peptides were predicted for 5 proteins in the F6 fraction, including oligopeptide and dipeptide-binding proteins. In Gram+ bacteria, lipoproteins are anchored in the cytoplasmic membrane and span the cell wall, but may also be released into the environment. The number of proteins without signal peptides were much more variable than those with signal peptides, ranging from 8 to 55 proteins in fraction F6 from strains A0901 and A1401, respectively (Fig. 5). All of these proteins were also detected in the WCL samples and included some of the most highly expressed intracellular proteins such as for example ribosomal proteins, RNA polymerase subunits and glyceraldehyde-3-phosphate dehydrogenase (Tables S4, S5). Finally, the proteomics analyses confirmed the expression of 13 phage proteins in *A. kunkeei* strain A0901, in agreement with the prediction of a prophage in the genome (Dyrhage et al. 2022) and the identification of phage particles in the nsTEM micrographs (Fig. 3B).

### The extracellular transcriptome

Transcriptomic data was collected using rRNA-depleted extraction protocols from the solubilized pellets obtained from the whole cell lysate and the cell free supernatant of *A. kunkeei* strain A1401. The CFP sample was highly concentrated after the ultracentrifugation step, such that it contained approximately 99% of the original cell suspension, while the WCL sample was prepared from only 1% of the growth media. Nevertheless, after the concentration and sample preparation steps there was no significant difference between the total number of reads obtained from the WCL and CFP samples either before or after rRNA depletion (p less than 0.05) (Table S7). In both samples, the rRNA reads were reduced to about 4% of the total reads after rRNA depletion, as compared to about 94% of the total reads before depletion (Table S7).

For comparison of the number of reads that mapped to protein-coding genes within each sample, TPM (transcripts per million) was used to estimate transcript abundance levels (Figure S8). After mapping and quantification of reads, only transcripts with TPM values ≥ 10 in either the CFP or the WCL group were included for further analysis, resulting in a data set that represented 1276 protein-coding genes (Table S8). For this set, the number of mapped reads per gene differed by more than three orders of magnitudes within each sample, ranging from a few genes associated with highly abundant transcripts with TPM values > 1000 to the large majority of genes with moderately abundant transcripts (100–1000 TPM) and lowly abundant transcripts with TPM values < 100.

**Figure 6.**
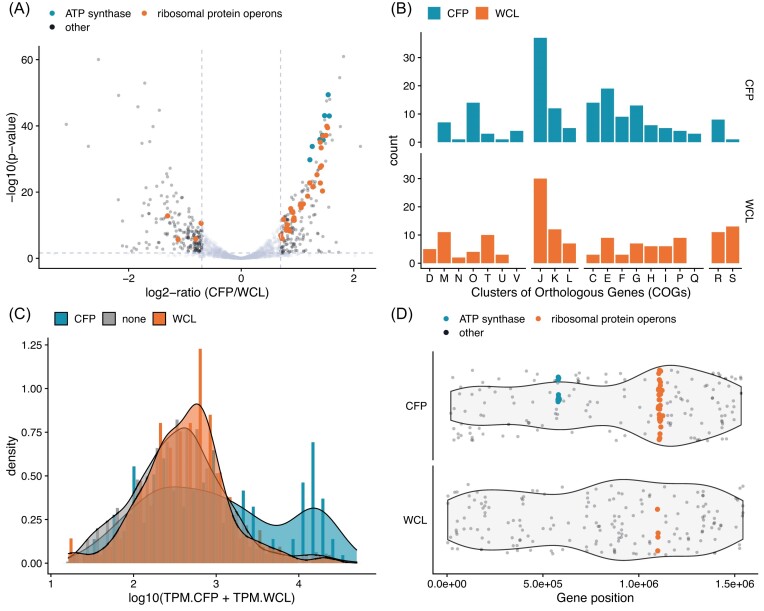
The whole-cell WCL and extracellular CFP transcriptome of *A. kunkeei* A1401. **(A)** Negative log10 p-values are shown as a function of the log2-ratio for the differentially enriched genes in the CFP and WCL fractions. Genes that are not classified to be enriched in either the WCL or CFP fraction are colored in light gray. Dashed vertical and horizontal lines indicate the thresholds for determining enrichment in either the CFP (positive log2-ratios) or WCL fraction (negative log2-ratios). **(B)** The frequencies of differentially enriched genes in the CFP and WCL transcriptomes are shown for the corresponding COG categories. Histograms and density plots illustrating the gene-frequency distribution as a function of the log10-transformed TPM-normalized read counts of the genes in the **(C)** CFP and **(D)** WCL fractions. Colors indicate enrichment in either the CFP (orange) or WCL fraction (blue) or whether genes are not enriched (‘none’, gray).

For comparisons of the relative transcript abundance levels in the CFP and WCL samples, DESeq2 analysis based on raw read counts was used (Fig. 6, Table S9). The results showed that 356 genes displayed differential enrichment patterns (adjusted p-values < 0.05, absolute log2-ratios ≥ 0.7), which corresponds to 28% of the analyzed gene set. Of these, 180 genes attracted relatively more reads from the CFP sample, while 176 genes were relatively more abundant in the WCL sample (Fig. 6A). The functional profiles of genes that showed no or differential enrichment were similar (Fig. 6B). However, the distribution of transcript abundances for the enriched genes in the WCL and CFP samples differed. While the enriched genes in the WCL sample showed a normal abundance distribution profile (Fig. 6C), the enriched genes in the CFP sample contained a higher fraction of the most abundant transcripts (Fig. 6D).

A more detailed comparison of the most abundant transcripts showed that they mapped to clusters of genes for ribosomal proteins and the ATP synthase complex (Figure S9). Ribosomal protein genes in the *spc* and alpha gene-clusters were highly enriched in the CFP sample and genes in the S10 gene-cluster were moderately enriched. In contrast, ribosomal protein genes in the upstream *str* operons were not enriched in either sample and genes for the ribosomal proteins RplM and RpsI, which were located elsewhere were enriched in the WCL sample. Beyond similar transcript abundance levels for genes that were clustered and likely part of the same operon, no other feature could be identified that accounted for the different enrichment profiles.

Finally, we used the transcriptomics data to gain an estimate of the relative abundances of transcripts for the secreted proteins with signal I peptides in the CFP sample (Table S6). Genes for the 33 kDa cysteine peptidase, the 37 kDa LPxTG cell wall binding protein and the 43 kDa matrixin import protein attracted a high abundance of reads, with TPM values in the range of 400–1000. The gene for the 158 kDa GH70 glycosyltransferase (A1401_12 750) recruited most reads with TPM values of 917 in the CFP sample and 1137 in the WCL sample, which supports the conclusion from the proteomics analysis that this is one of the most highly abundant proteins in the CFP sample.

## Discussion

Extracellular MVs have been described in both Gram-negative and Gram-positive bacteria, archaea and eukaryotes, and numerous reports have suggested that they serve various functional roles in for example cell-cell communication processes, host adaptation and antimicrobial defense by mediating the transfer of RNA, proteins and metabolites across cell boundaries (Brown et al. 2015, Liu et al. 2018, Gill et al. 2019, Yu et al. 2022). An alternative hypothesis, particularly for eukaryotic MVs, is that their basic function is to secrete material out from the cell for off-site degradation and recycling of membrane molecules (Vidal 2019). Here, we report the results from a broad survey of the RNA and protein cargo of the secreted material from *A. kunkeei*, a defensive symbiont of honeybees. The starting point for our analyses was the observation that *A. kunkeei* strain A1401 contained surface-associated structures on the bacterial cells. For comparison, we also included strain A0901 which seemed to have fewer such structures. However, analyses by TEM, proteomics and other techniques showed no major differences of the extracellular material produced by the two strains. Based on distinct morphological and biochemical features of the extracellular particles, we infer that they represent two different types; membrane vesicles and a large proportion of non-membranous protein particles of 30–40 nm in size.

Before discussing the contents of the secreted molecules, let us first briefly comment on the methods used for preparation of the particles. Various isolation protocols have been used in the past to examine the content of MVs, and these can roughly be categorized into ultracentrifugation-, filtration-, aggregation- and affinity-based approaches. Approaches based on ultracentrifugation have most commonly been used, sometimes combined with differential gradient ultracentrifugation to separate the targeted particles primarily from loosely bound proteins (Konoshenko et al. 2018). Our and many other protocols include a low-speed centrifugation to deplete the cell suspension from large debris and intact cells followed by filtration through 0.22–0.45 μm filters to completely remove residual cells and debris. Growth control is important at this stage to ensure that the filtrate is cell-free. Following filtration, the secreted particles in the cell-free supernatant were pelleted by ultracentrifugation to yield what we refer to as cell-free pellet (CFP). Omics and imaging analysis were performed on the crude CFP, essentially as described before for other *Lactobacillus* species (Dean et al. 2019, Hu et al. 2021).

We used a combination of electron microscopy, including SEM, TEM, nsTEM and cryo-ET, to obtain a comprehensive and detailed view on the morphology of isolated protein particles and MVs from *A. kunkeei*. For biophysical analysis, we used Nanoparticle Tracking Analysis (NTA), commonly employed for characterizing exosomes and membrane vesicles. We observed a discrepancy between the size distributions obtained from TEM, nsTEM and cryo-ET data in comparison to the NTA data, where the size appeared to be overestimated by NTA. This has been observed before (Bachurski et al. 2019) and might be the result of the hydrodynamic radius measured by NTA being larger than the actual particle size. It has also been noted that the detection limit for NTA is 60–70 nm (Bachurski et al. 2019), which is larger than the speckled particles and small MVs that we clearly detected by electron microscopy. This can either indicate that, in the polydisperse and heterogenous CFP, NTA detects aggregated particles and MVs, or that the hydrodynamic radius of the isolated particles is larger than the actual particle radius. Despite those uncertainties, the possibility to obtain an estimate of the particle concentration makes NTA a valuable method for quality control, and in the present study particle concentration correlated very well the protein concentration. In isolated particle preparations, they were observed as single particles as well as associated to form smaller chains. This was also observed in tomograms of whole bacterial cells indicating that chain formation is a biologically relevant process and not an experimental artefact due to aggregation upon ultracentrifugation.

For more detailed analyses of the CFP proteomes and transcriptomes, we also employed density gradient ultracentrifugation using an Optiprep gradient, similarly as described for the isolation of bacterial and plasma extracellular vesicles (Karimi et al. 2018, Onódi et al. 2018). In those previous and our studies, the crude CFP-preparation was loaded on top of the density gradient. In other studies where bacterial membrane vesicles were isolated in an Optiprep gradient, the crude pellet was re-suspended in the highest density solution, loaded in the bottom of the centrifuge tube and overlayered with decreasing Optiprep concentrations (Dauros Singorenko et al. 2017, Dean et al. 2020, Rodriguez and Kuehn 2020, Hu et al. 2021). Such an approach is based on the flotation of membrane vesicles against the centrifugal force until the vesicles reach the corresponding density, while loose proteins, due to their high density, will stay pelleted at the bottom of the tube (Graham 2002). Choosing the top-loading approach seemed more rational for our purpose as we observed a novel and apparently non-membranous type of secreted particles in the crude CFP, together with MVs, without *a priori* information on the corresponding particle densities.

The results of the DGUC-fractionation provided further evidence that the secreted material consists of distinct particle types with different physicochemical properties. Membrane-rich particles were enriched at a density of approximately 1.13 g/mL, peaking in DGUC fraction F3. Negative stain TEM analysis of this fraction demonstrated the presence of typical membrane vesicles of variable sizes in addition to large mesh-like structures. The membrane-less and speckled particles, representing the majority of detected particles in the crude CFP-preparation were separated at a density of 1.19 g/mL, peaking in DGUC fractions F6 and F7. The RNA showed a similar fractionation pattern as the membranes with a peak in DGUC fraction F3, while the proteins showed a similar fractionation pattern as the speckled particles with a peak in DGUC fractions F6 and F7. A comparison of the protein composition in the crude CFP samples and DGUC fractionated samples, as inferred from the intensities and sizes of the bands on an SDS-PAGE gel as well as from analyses by MS/MS-LC, suggest that all fractions contain a similar set of proteins, albeit in different abundances. Based on this observation, we conclude that the large majority of proteins identified in the CFP sample fractionated with the particles, rather than with the MVs. However, we considered the possibility that a less abundant subset of the CFP proteins, which would go undetected on the SDS-PAGE gel, were associated with the MVs in DGUC fraction F3. To test this hypothesis, we analyzed and compared the protein compositions of fractions F3 and F6, but were unable to identify proteins uniquely associated with fraction F3 in both strains. This suggests that the MVs produced by the *A. kunkeei* cells do not package a specific subset of proteins.

A comparison of the CFP proteomes showed that 50 proteins were present in three biological replicates in both strains, about half of which contained type-I signal peptides and are thus likely to correspond to secreted proteins in *A. kunkeei*. The CFP proteomes contained most of the 24 proteins identified in a previous study of the cell-free supernatant of *A. kunkeei* strain Fhon2 under LPS-stress (Butler et al. 2013). The core CFP proteins included the giant proteins, which are 3 000 to 9 000 amino acids in length and encoded by a 90 kb long cluster of genes in the *A. kunkeei* genome (Tamarit et al. 2015). All five giant proteins were identified in strain A0901, and the proteins encoded by the first two genes in the cluster were also detected in strain A1401. Three of the proteins contain transmembrane domains, two of which were also identified in the WCL sample, suggesting that the giant proteins are surface-associated. Their function is unknown but like other very large extracellular surface proteins, they may be involved in surface adherence.

The identified core CFP proteins additionally included large membrane-associated glycoside hydrolases of 114 to 158 kDa, which are likely to serve important functions in the synthesis or modification of polysaccharides such as dextran. In gel-digestion confirmed that the high molecular weight glycosyltransferases represent some of the most highly abundant proteins in the CFP samples. In addition to the type-I signal peptide, these enzymes also contained the KxYKxGKxW signal peptide, which is a characteristic signal sequence of serine-rich, large glycosylated surface adhesins in Gram-positive bacteria (Gagic et al. 2013). One of the glycosyltransferases (A1401_12750) showed sequence similarity to the *A. kunkeei* enzyme GtsZ, which contains two catalytic cores, CD1 and CD2, predicted to have glucansucrase and branching sucrose specificity, respectively (Meng et al. 2018). Biochemical studies of GtsZ-CD2 have confirmed the predicted branching sucrose specificity and showed that this protein catalyzes the production of structurally complex alpha-glucans (Meng et al. 2018). However, GtsZ-CD1 could not be expressed and was therefore not characterized further. Interestingly, the two catalytic cores are split into two genes in strain A1401, of which the protein encoded by A1401_12750 corresponds to GtsZ-CD1, whereas A1401_12760, which corresponds to GtsZ-CD2 could not be detected in neither the CFP nor the WCL proteome. Consistently, the TPM values for A1401_12760 corresponded to only 1%–2% of the TPM values for A1401_12750, which attracted a high abundance of RNA reads in both the CFP and the WCL samples. The demonstrated high abundance of GtsZ-CD1 in this strain now enables a more detailed biochemical study of the glucan sucrase activity.

Proteomic studies on membrane vesicles from other bacterial species have shown release of cytoplasmic proteins without predicted signal peptides or other export signals. It should be noted though that several of those studies did not compare the extracellular proteome to the whole-cell proteome, which is important in order to conclude whether a protein is uniquely present or enriched in the extracellular milieu or simply present as a result of the detection of trace amounts of a highly expressed protein, such as EF-Tu. In our dataset, EF-Tu was reliably detected in both the WCL and CFP samples, as were also ribosomal proteins, RNA polymerase subunits and ATP synthase subunits. However, in contrast to the consistent detection of the same set proteins with signal peptides in multiple extractions from two different strains, the identification of cytoplasmic proteins was more variable and the results were not reproducible between either strains or samples. Moreover, it should be recalled that the CFP samples are highly concentrated compared to the WCL samples, and it can therefore not be excluded that intracellular proteins derived from a small fraction of lysed cells may be detected in the CFP samples.

Importantly, our study indicated that the secreted proteins were not embedded within the MVs, which instead showed the same fractionation pattern as the RNA molecules. We used a rRNA depletion protocol, which depleted more than 95% of the rRNA pool of both CFP and WCL samples but also prevented any comparative analysis based on rRNA abundances. We detected tRNA sequences in our data, but comparative analyses of tRNA sequencing data are hampered by the modifications and rigid structures of these molecules. Accurate estimates of tRNA levels in the cells would have required targeted library preparation protocols, which was not employed in this study (Schwartz et al. 2018, Pinkard et al. 2020). Thus, although we recovered different types of RNA from the total RNA pool of both the CFP and WCL fractions, including rRNA and tRNA, the subsequent analyses were focused on the mRNA transcripts.

Overall, we estimated that the total amount of secreted RNA molecules represented no more than about 1% of the amount of cellular RNA, and is thus only a tiny fraction of the intracellular RNA. Unlike the secreted proteins which were mostly uniquely present in the extracellular material, all transcripts detected in the CFP sample were also identified in the WCL sample. Sequence read coverage for each data set varied by several orders of magnitude for the individual protein-coding genes. However, we noted a relatively higher abundance of RNA fragments for the most highly expressed genes in the CFP samples compared to the WCL samples, such as those coding for ribosomal proteins, RNA polymerase subunits and ATP synthase subunits. Likewise, a biased composition of the RNA content of the MVs were observed in *S. aureus* (Luz et al. 2021). As in our study, the RNA transcripts covered co-located genes, presumably belonging to the same operons, which code for highly expressed genes involved in translation, energy production and carbohydrate metabolism, but the previous study showed that the compositional bias depended on the environmental conditions (Luz et al. 2021).

This leaves open the question of the biological role that the extracellular RNA sequences play, if any. One hypothesis is that the transfer of functional RNA to recipient cells that are lacking a corresponding gene might confer a transient horizontal phenotype (Luz et al. 2021), which seems unlikely in *A. kunkeei* since the most abundant transcripts in the CFP samples were obtained from the universal core genes. Another hypothesis is that the secreted RNA molecules mediate bacterial interactions with the host, such as for example serving host immunomodulatory functions (Tsatsaronis et al. 2018, Rodriguez and Kuehn 2020, Kurata et al. 2022). While small, non-coding RNAs have been directly associated with immunomodulatory responses (Koeppen et al. 2016, Yu et al. 2022, Sahr et al. 2022), the specificity of such effects by bulk-RNA are not fully resolved. Furthermore, previous studies have shown that RNAs are secreted as fragments, arguing against a biological role for the large majority of secreted RNA molecules derived from long mRNA molecules (Rodriguez and Kuehn 2020, Luz et al. 2021, Kurata et al. 2022). Selective explanations also require some mechanism whereby the RNAs that modulate the proposed functions in the recipient cells are selected for secretion and packaging, however, no such targeting sequencing or selective packaging mechanism have yet been identified.

We acknowledge the possibility that the mRNAs in the CFP samples may be randomly associated with or embedded inside the MVs, for example upon cell lysis, like the intracellular proteins. If so, the observed differences in the composition of the RNA cargo in the CFPs compared to WCL samples might be due to a relatively higher recovery of mRNAs with long half-lives in the CFP sample and/or to the use of different protocols for preparation of RNA from the two samples. However, even if the RNA fragments associated with the MVs would result from lysis of a small fraction of bacterial cells, this does not exclude the possibility that some of the short RNA fragments may have evolved to serve specific roles in specific recipient cells. It remains to be shown in future studies whether the mRNA molecules identified in the CFP samples of *A. kunkeei* are involved in interaction and communication processes among the bacterial cells or with the honeybees. In particular, the secretion and putative role of small RNAs in *A. kunkeei* need further investigation.

In summary, we have demonstrated the presence of distinct extracellular particle types in two *Apilactobacillus kunkeei* strains. The analyses suggest that the CFP fraction consists of secreted protein complexes, cell surface proteins and phage proteins as well as MVs that are associated with cellular RNA. To the best of our knowledge, this is the first time that the RNA and protein content of extracellular samples from a bacterial source have been separated into two biochemically and morphologically distinct fractions. So, although both MVs, RNA and protein particles may be part of the same extracellular material, they may not necessarily be associated with each other. Future studies should be directed towards elucidating the structure, function and evolution of the surface-associated and secreted proteins, particularly the giant proteins and the glycoside hydrolases. Possible functional roles could involve a defensive mechanism by the secreted peptidases and GH25 hydrolases and, at the same time, the production of an exopolysaccharide layer by the action of the GH70 hydrolases.

## Supplementary Material

uqad037_Supplemental_Files

## Data Availability

The mass spectrometry proteomics data have been deposited to the ProteomeXchange Consortium via the PRIDE (Perez-Riverol et al. 2022) partner repository with the dataset identifier PXD034473 (*A. kunkeei* strain A1401) and PXD034474 (*A. kunkeei* strain A0901). The transcriptomic data discussed in this publication have been deposited in NCBI's Gene Expression Omnibus (Edgar et al. 2002, Barrett et al. 2013) and are accessible through GEO Series accession number GSE205998 (https://www.ncbi.nlm.nih.gov/geo/query/acc.cgi?acc= GSE205998). Reconstructed cryo-electron tomograms have been deposited to the EMDB (Lawson et al. 2016) database (see Table S10 for EMDB Accession IDs). TEM, SEM and nsTEM micrographs have been deposited to the SciLifeLab Data Repository under the DOI 10.17044/scilifelab.c.6232599.
