# Supplementary material for: Apilactobacillus kunkeei releases RNA-associated membrane vesicles and proteinaceous nanoparticles
Source: Microlife. 2023 Aug 29;4:uqad037. doi: 10.1093/femsml/uqad037 (PMC10496945; doi:10.1093/femsml/uqad037)
Supplement: uqad037_Supplemental_Files [file uqad037_supplemental_files.zip › Supplementary.Figures.R2.docx]

10 Supplementary Material

# Supplementary Figures and Tables

## Supplementary Figures

**Supplementary Figure S1.** Particle, total protein and total RNA concentration of isolated cell-free pellets (CFP) from *A. kunkeei* strains A0901 and A1401. The estimated cell concentration is indicated as CFU [CFU/mL]. Mean and standard deviations from three biological replicates are shown for each strain.

**Supplementary Figure S2.** Analysis of particle distribution by Nanoparticle Tracking Analysis. Crude CFP preparations of *A. kunkeei* strains A1401 and A0901 were analyzed by NTA. The estimated concentration (in particles/mL) is shown as a function of the estimated size (in nm). The sizes of the major peaks are indicated in each panel.

**Supplementary Figure S3.** Nuclease protection assay of the crude CFP preparations of *A. kunkeei* strains A0901 and A1401. (A) Total RNA concentration was measured by the Qubit RNA HS assay of untreated CFP preparations (“C”) and preparations subjected to complete lysis by proteinase K and SDS (“PS”). No significant differences in total RNA concentration were found between untreated and treated CFP preparations (p<0.05, unpaired T-Test with Welch’s correction). After RNAse treatment, the levels of total RNA decreased below the detection limit for all measured samples.

**Supplementary Figure S4.** Analysis of density-gradient ultracentrifugation fractions obtained from separated crude CFP samples of *A. kunkeei* A1401. Fractions F1–F10 (increasing density), were analysed for particle concentration (NTA), protein content (Bradford), RNA (Qubit RNA HS assay, SYTO RNASelect) and membranes (FM4-64). Results are presented as mean and standard deviation based on 3 biological replicates.


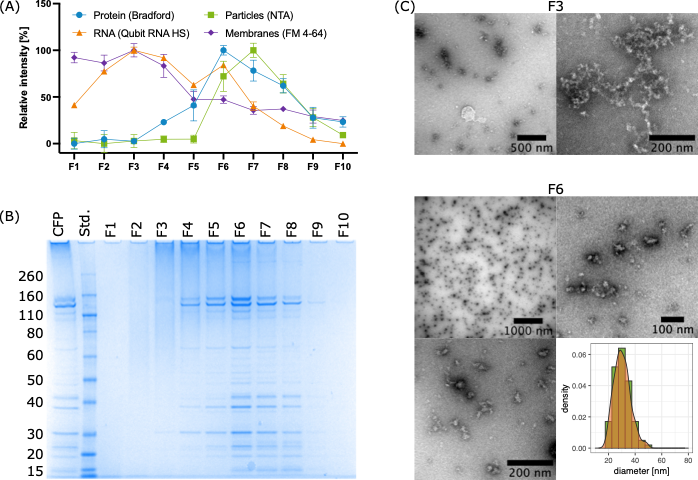


**Supplementary Figure S5.** Separation of CFP components by density-gradient ultracentrifugation (DGUC). Crude CFP samples (n = 3, biological replicates) from *A. kunkeei* strain A0901 were separated by Optiprep-based DGUC into 10 fractions. (A) Analysis of DGUC fraction for particles (NTA), protein (Bradford), RNA (Qubit RNA HS assay, SYTO RNASelect) and membranes (FM4-64). Relative concentrations and intensities are shown based on average and standard deviation from three biological replicates. (B) SDS-PAGE analysis of DGUC fractions (F1–F10) of representative replicate sample. The crude CFP sample (10x diluted) was loaded for comparison with the DGUC fractions. Novex Sharp Unstained marker was used as the molecular weight standard and the corresponding molecular weights (in kDa) are shown next to the gel. (C) Negative stain TEM analysis of DGUC fractions F3 and F6. The size distribution is based on manual measurements of particles performed in Fiji/ImageJ.

**Supplementary Figure S6.** SDS-PAGE analysis. WCL and CFP fractions isolated from *A. kunkeei* strain (A) A1401 and (B) A0901. The molecular weight (in kDa) of the protein standard ("Std.") is indicated next to the gel. (C) SDS-PAGE analysis of fMRS media control. Lane 1: fMRS media control (15.5 µL); Lane 2: protein standard (molecular weights in kDa); Lanes 3–5: fMRS media control after ultracentrifugation (1.6 µL). Lane 6: fMRS media control (15.5 µL); Lane 7–9: fMRS media control after ultracentrifugation (3.2 µL); Lane 10–12: fMRS media control after ultracentrifugation (15.6 µL).

**Supplementary Figure S7.** SDS-PAGE analysis of DGUC fractions of cell-free pellets from *A. kunkeei* strain A1401 and A0901. Lane 1: Cell-free pellet (1.6 µL, A1401), fMRS media control (15.5 µL A0901); Lane 2: protein standard (molecular weights in kDa); Lanes 3–12: DGUC fractions 1–10 (15.6 µL).

**Supplementary Figure S8. RNA read count distribution in *A. kunkeei* A1401 CFP and WCL fractions.** Histograms based on log10-transformed TPM-normalized read counts illustrate the read-count distribution of the (A) CFP and (B) WCL fractions isolated from *A. kunkeei* A1401.

**Supplementary Figure S9. Gene order structures for ribosomal protein and ATP synthase subunits**. Genes are colored according to the log2-ratio from the comparison of the CFP and WCL fractions based on transcriptomic analysis. Positive values (colored in red) versus negative values (colored in blue) indicate that a relatively higher level of transcripts was detected in the CFP versus the WCL fractions.

## Supplementary Tables

**Supplementary Table S1.** Summary of size estimates of secreted particles based on electron microscopy. The median, minimum and maximum particle diameters for the detected secreted particles are summarized for the respective *A. kunkeei* strain. The corresponding electron microscopy technique, sample and particle type are indicated as well as the number of measured particles.

**Supplementary Table S2.** Summary size distributions and particle concentrations of secreted particles based on NTA. Average and standard deviation from three biological replicates per strain are summarised for particle concentration, mode peak of the distribution, OD600 of the culture, corresponding cell concentration, particles in the culture per mL cell suspension and the ratio of particles to cell number.

**Supplementary Table S3.** Protein concentrations as determined by the Bradford assay for DGUC fractions subjected to proteomic analysis after in-solution digestion. Asterisks indicate protein concentrations that were below the limit of detection.

**Supplementary Table S4.** Combined proteomic results for *A. kunkeei* strains A1401 and A0901. The included proteins are identified in 3 replicates in at least one of the sample groups (CFP: LFQ intensity 4–6; WCL: LFQ intensity 1–3)*.* Additional relevant columns: *SignalP5.0*: signal peptide prediction by SignalP5.0; *TM* and *SP*: predicted transmembrane domains and signal peptides by Phobius; *cog* and *cog.family*: functional prediction based on Clusters of Orthologous Genes; *position*: center position of gene on chromosome; *Fhon2.locus.tag*: locus-tag of homologs that have been identified in the cell-free supernatant in *A. kunkeei* strain fhon2 (Butler et al., 2013). Corresponding ID of fhon2-homologs in comparative genomic analysis of *A. kunkeei* (Tamarit et al., 2015); *ortho:* OrthoMCL cluster; *strain*: *A. kunkeei* strain.

**Supplementary Table S5.** Combined proteomic results for *A. kunkeei* strains A1401 and A0901 after Density Gradient Ultracentrifugation. The included proteins are identified in 3 replicates in at least one of the sample groups (CFP or WCL)*.* Additional relevant columns: *SignalP5.0*: signal peptide prediction by SignalP5.0; *TM* and *SP*: predicted transmembrane domains and signal peptides by Phobius; *cog* and *cog.family*: functional prediction based on Clusters of Orthologous Genes; *position*: center position of gene on chromosome; *sample*: sample type (CFP-, F3-, F6-sol: in-solution digested samples; F6-gel: in-gel digested samples) strain: *A. kunkeei* strain; *n_detected*: number of biological replicates a protein was detected in; *replicate*: number of replicate (1–3).

**Supplementary Table S6.** Summary of proteomics results for protein families with at least one member found in all replicates in either CFP, F3, or F6. The CFP, F3, and F6 columns show the number of replicates in which the given protein was detected in for each sample. Additional relevant columns: orthogroup: OrthoMCL cluster; cog: functional prediction based on Clusters of Orthologous Genes; SignalP5.0: signal peptide prediction by SignalP5.0; TM and SP: predicted transmembrane domains and signal peptides by Phobius; Fhon2.locus.tag: locus-tag of homologs that have been identified in the cell-free supernatant in *A. kunkeei* strain fhon2 (Butler et al., 2013). Corresponding ID of fhon2-homologs in comparative genomic analysis of *A. kunkeei* (Tamarit et al., 2015).

**Supplementary Table S7.** Number of read counts in CFP (*MV1–3*) and WCL (*WC1–3*) samples before and after rRNA depletion.

**Supplementary Table S8.** Summary of results from transcriptome analysis of *A. kunkeei* A1401 for comparison of CFP and WCL samples. The *locus.tag*, chromosome (*Chr*), *Start* and *End* position of the genes and *gene.length* (gene length in nucleotides, nt) are listed for each gene together with raw read counts from featurecounts (*CFP1–3*, *WCL1–3*) and TPM-corrected read counts (*CFP1–3.TPM*, *WCL1–3.TPM*).

**Supplementary Table S9.** Summary of results from transcriptome analysis of *A. kunkeei* A1401 for comparison of CFP and WCL samples. *log2FoldChange*, *pvalue*, *padj*: log2-ratio, p-value and adjusted p-value from DESeq2 analysis of CFP and WCL groups; *gene.length*: gene length (nt) of respective genes; *CFP1–3.DESeq2*, *WCL1–3.DESeq2*: normalised read counts obtained from DESeq2 analysis; *DGE.rna*: enrichment in CFP or WCL groups or none based on DESeq2 analysis; *position*: center position of gene on chromosome; *cog* and *cog.family*: functional prediction based on COG; *SignalP5.0*: signal peptide prediction by SignalP5.0; *phobius.TM* and *phobius.SP*: predicted transmembrane domains and signal peptides by Phobius; *meta*: additional information on genes, like operons or operon clusters.

**Supplementary Table S10.** Summary of reconstructed cryo-ET tomograms. Cryo-ET tomograms were reconstructed for isolated ECPs of *A. kunkeei* A1401 and whole-cells obtained during log-phase. *Accession-ID* indicates the EMDB ID for each of the reconstructed *tilt.series*.

# Supplementary References

Butler, È., Alsterfjord, M., Olofsson, T. C., Karlsson, C., Malmström, J., and Vásquez, A. (2013). Proteins of novel lactic acid bacteria from Apis mellifera mellifera: an insight into the production of known extra-cellular proteins during microbial stress. *BMC Microbiol* 13, 1–11. doi: 10.1186/1471-2180-13-235.

Tamarit, D., Ellegaard, K. M., Wikander, J., Olofsson, T., Vásquez, A., and Andersson, S. G. E. (2015). Functionally Structured Genomes in Lactobacillus kunkeei Colonizing the Honey Crop and Food Products of Honeybees and Stingless Bees. *Genome Biol Evol* 7, 1455–1473. doi: 10.1093/gbe/evv079.
